# Exosomes Derived from CXCR4-Overexpressing BMSC Promoted Activation of Microvascular Endothelial Cells in Cerebral Ischemia/Reperfusion Injury

**DOI:** 10.1155/2020/8814239

**Published:** 2020-12-18

**Authors:** Xutong Li, Ye Zhang, Yong Wang, Dan Zhao, Chengcheng Sun, Shaoting Zhou, Dongsheng Xu, Jing Zhao

**Affiliations:** ^1^Department of Neurology, Minhang Hospital, Fudan University, Shanghai, China; ^2^Department of Rehabilitation, Tongji Hospital, Tongji University, Shanghai, China; ^3^Department of Rehabilitation, Dongfang Hospital, Tongji University, Shanghai, China; ^4^Rehabilitation Center, Yueyang Hospital of Integrated Traditional Chinese and Western Medicine, Shanghai, China; ^5^College of Rehabilitation Science, Shanghai University of Traditional Chinese Medicine, Shanghai, China; ^6^Engineering Research Center of Traditional Chinese Medicine Intelligent Rehabilitation, Ministry of Education, Shanghai, China

## Abstract

**Background:**

Ischemic stroke is a severe acute cerebrovascular disease which can be improved with neuroprotective therapies at an early stage. However, due to the lack of effective neuroprotective drugs, most stroke patients have varying degrees of long-term disability. In the present study, we investigated the role of exosomes derived from CXCR4-overexpressing BMSCs in restoring vascular function and neural repair after ischemic cerebral infarction.

**Methods:**

BMSCs were transfected with lentivirus encoded by CXCR4 (BMSC^CXCR4^). Exosomes derived from BMSC^CXCR4^ (Exo^CXCR4^) were isolated and characterized by transmission electron microscopy and dynamic light scattering. Western blot and qPCR were used to analyze the expression of CXCR4 in BMSCs and exosomes. The acute middle cerebral artery occlusion (MCAO) model was prepared, Exo^CXCR4^ were injected into the rats, and behavioral changes were analyzed. The role of Exo^CXCR4^ in promoting the proliferation and tube formation for angiogenesis and protecting brain endothelial cells was determined *in vitro*.

**Results:**

Compared with the control groups, the Exo^CXCR4^ group showed a significantly lower mNSS score at 7 d, 14 d, and 21 d after ischemia/reperfusion (*P* < 0.05). The bEnd.3 cells in the Exo^CXCR4^ group have stronger proliferation ability than other groups (*P* < 0.05), while the CXCR4 inhibitor can reduce this effect. Exosomes control (Exo^Con^) can significantly promote the migration of bEnd.3 cells (*P* < 0.05), while there was no significant difference between the Exo^CXCR4^ and Exo^Con^ groups (*P* > 0.05). Exo^CXCR4^ can further promote the proliferation and tube formation for the angiogenesis of the endothelium compared with Exo^Con^ group (*P* < 0.05). In addition, cobalt chloride (COCl2) can increase the expression of *β*-catenin and Wnt-3, while Exo^Con^ can reduce the expression of these proteins (*P* < 0.05). Exo^CXCR4^ can further attenuate the activation of Wnt-3a/*β*-catenin pathway (*P* < 0.05).

**Conclusions:**

In ischemia/reperfusion injury, Exo^CXCR4^ promoted the proliferation and tube formation of microvascular endothelial cells and play an antiapoptotic role via the Wnt-3a/*β*-catenin pathway.

## 1. Introduction

Stroke is an acute cerebrovascular disease caused by local cerebral blood circulation disorder, which is the second cause of global human death [1]. According to the report published by the World Health Organization in 2017, about 6.24 million people die of stroke every year [2]. Among the surviving stroke patients, over 70% of them have varying degrees of long-term disability [3]. Stroke includes ischemic and hemorrhagic stroke, and the former is the most common subtype, accounting for 87% of all cerebrovascular accidents [4]. Currently, intravenous alteplase and mechanical thrombectomy are recommended effective treatments for patients with acute ischemic stroke. However, these two treatments have many limitations, especially the narrow time window, which largely limits their clinical use [5]. Thus, it is necessary to find alternative therapeutics for patients with acute ischemic stroke.

The brain microvascular endothelium is a thin layer of connected and anchorage-dependent cells, which constitutes the interface between the bloodstream and the deformable solid vascular wall [6]. This vascular endothelium is a highly active metabolic system that synthesizes various vascular regulatory factors to adjust the microcirculation of the cerebral tissue [7]. The brain microvascular endothelium cells are now recognized as a highly active metabolic system that synthesizes various materials to nourish nerves and regulate vasomotor function [8]. As is well known, effective collateral circulation can protect brain tissue after cerebral ischemia by increasing blood perfusion in the ischemic penumbra (IP) [9]. It has been demonstrated that microvascular integrity and cerebrovascular angiogenesis may stabilize brain perfusion thereby promoting neuronal survival, brain plasticity, and neurologic recovery [10–13]. Thus, enhancing angiogenesis in ischemic brain tissue is considered to be an emerging opportunity for potential therapeutic strategies.

Bone marrow mesenchymal stem cells (BMSCs) are able to secrete a variety of trophic factors promoting cell repair and growth [14]. Many studies indicated that stem cells can inhibit inflammation and promote angiogenesis in the ischemic area. Therefore, stem cell therapy is considered to be one of the most promising treatments for ischemic stroke [15, 16]. CXC motif chemokine receptor type 4 (CXCR4) belongs to G-protein-coupled receptor superfamily, which is involved in the homing of a variety of cells. The ligand of CXCR4 is stromal cell-derived factor 1 (SDF-1 or CXCL12) belonging to CXC chemokine family. The expression of CXCL12 is significantly increased in ischemic brain tissue [17] because of the activation of hypoxia-inducible factor-1 (HIF-1). Previous studies have shown that plasma CXCL12 will increase in poststroke patients, which indicated that the concentration gradient of CXCL12 will form in the blood when it is released from the ischemic area [18, 19]. To verify the angiogenic roles of CXCL12 *in vivo*, our previous study utilized the chicken chorioallantoic membrane (CAM) assay. We found that CXCL12 in doses of 50 and 100 ng/ml induced neovessel formation, which indicated that CXCL12 enhanced the capacity for angiogenesis *in vivo* [20]. CXCL12 can bind to the CXCR4 on CXCR4-expressing cells, including brain microvascular endothelial cells (EC). This promotes the migration of cerebral microvascular endothelial cells to ischemic tissues improving the brain tissue repair. Vessel sprouting demands migration and polarization of ECs in response to cytokines [21]. Our previous study indicated that CXCR4 may be involved in the migration and polarization of human umbilical vein endothelial cells in the stripe assay [20]. Collectively, these results are the foundation of the present study to investigate the role of CXCR4 in promoting angiogenesis and protecting brain endothelial cells.

Exosomes are small cell membrane vesicles with a diameter of 30-100 nm; the contents are lipids, proteins, RNA, miRNAs, and mRNAs [22, 23]. Exosomes are carriers of intercellular signal transmission, playing an important role in intercellular communication. The improvement of neural function depends on the entire neurovascular unit, including neurons, astrocytes, vascular endothelial cells, basement membrane, and extracellular matrix. A signal transduction network between cells plays an important role in tissue reconstruction after cerebral ischemia [24]. Increasing evidence is demonstrating that exosomes of mesenchymal stem cells can promote vascular remodeling in the ischemic zone after cerebral ischemia [25]. However, whether the exosomes deprived from CXCR4-overexpressing BMSCs play a role in the angiogenesis is still unclear.

In the present study, we hypothesized that exosomes derived from BMSCs could effectively regulate cell survival, promote angiogenesis, protect nerve cells, and improve stroke outcome in the acute stage of stroke, through upregulating CXCR4 in exosomes of BMSCs. We aim to investigate the role of exosomes derived from CXCR4-overexpressing BMSCs in restoring vascular function and neural repair after ischemic cerebral infarction and to clarify the mechanism of these exosomes in promoting angiogenesis and protecting endothelial cells, so as to provide new ideas for the treatments and further mechanism research of stroke.

## 2. Materials and Methods

### 2.1. Chemicals and Reagents

The chemicals and reagents used in the present study include RIPA lysis buffer (Beyotime Biotechnology, Shanghai), bicinchoninic acid assay (BCA) protein assay kit (Beyotime Biotechnology, Shanghai), sodium dodecyl sulfate-polyacrylamide gel electrophoresis (SDS-PAGE; Beyotime Biotechnology, Shanghai), enhanced chemiluminescence (ECL; Beyotime Biotechnology, Shanghai), 4′,6-diamidino-2-phenylindole (DAPI; Thermo, USA), protein marker (Thermo, USA), Tris-buffered saline tween (TBST; Solarbio, Beijing), phosphate-buffered saline (PBS; Solarbio, Beijing), methanol (Sinopharm, Beijing), anhydrous ethanol (Sinopharm, Beijing), pentobarbital (Sinopharm, Beijing), Dulbecco's modified Eagle medium (DMEM; Gibco, USA), PHK26 (Sigma, USA), 6-0 coated with silicon nylon monofilament suture (Beijing Cinontech Co., Ltd., Beijing), fetal bovine serum (FBS; Beyotime Biotechnology, Shanghai), normal saline (NS; Beyotime Biotechnology, Shanghai), Trizol reagent (Invitrogen, United States), the PrimeScript™ RT reagent Kit (TAKARA, Japan), SYBR Premix Ex Taq™II (TAKARA, Japan), rabbit anti-SQSTM1/p62 (D1Q5S) antibody (1 : 1000 dilution; CST, Boston), rabbit anti-LC3A/B (D3U4C) XP antibody (1 : 1000 dilution; CST, Boston), rabbit anti-BAX antibody (1 : 1000 dilution; Abcam, Cambridge, MA), goat anti-CXCR4 (1 : 1000 dilution; Abcam, Cambridge, MA) antibody, mouse anti-*β*-tubulin (1 : 1000 dilution; Sigma, USA) antibody, rabbit anti-TSG101 (1 : 1000 dilution; CST, Boston) antibody, rabbit anti-CD9 (1 : 1000 dilution; Abcam, Cambridge, MA) antibody, mouse anti-Alix (1 : 1000 dilution; CST, Boston) antibody, mouse anti-Bcl2 (1 : 1000 dilution; Abcam, Cambridge, MA) antibody, rabbit anti-PARP (1 : 1000 dilution; CST, Boston) antibody, rabbit anti-Wnt-3a (1 : 1000 dilution; Wanleibio, Shenyang) antibody, rabbit anti-*β*-catenin (1 : 1000 dilution; Millipore, Billerica, MA) antibody, mouse anti-*β*-actin (1 : 1000 dilution; Proteintech, Chicago) antibody, rabbit anti-CD105 (BD biosciences, San Diego) antibody, rabbit anti-CD44 (BD biosciences, San Diego) antibody, rabbit anti-CD73 (BD biosciences, San Diego) antibody, and rabbit anti-CD90 (BD biosciences, San Diego) antibody.

### 2.2. Cell Culture

The BMSCs were taken from the bone marrow cavity of rat femur. Sprague-Dawley (SD) rats weighing 200 g were anesthetized with excessive pentobarbital intraperitoneally, and the femurs on both sides were removed. The femoral bone marrow cavity was washed repeatedly with low-glucose complete medium to prepare cell suspension, and the primary cells were cultured in a low-glucose DMEM (Gibco, USA) supplemented with 10% FBS. The 3rd to 6th generation BMSCs were used in the following experiments. Mouse brain microvascular endothelial cells (bEnd.3) were purchased from the American Type Strain Collection Center (ATCC CRL-2299). bEnd.3 cells were cultured in DMEM (Gibco, USA) supplemented with 10% FBS in a 5% CO_2_-humidified atmosphere at 37°C. All animal studies were approved by the Institutional Animal Care and Use Committee (IACUC) of Shanghai Fu Dan University, Shanghai, China.

### 2.3. Flow Cytometry

BMSCs were washed twice with PBS to collect 1 to 5 × 10^5^ cells. 1 × binding buffer was added to resuspend cells, and the cell concentration was adjusted to 10^6^/ml cells. Antibodies of CD105 (BD biosciences, San Diego), CD44 (BD biosciences, San Diego), CD73 (BD biosciences, San Diego), and CD90 (BD biosciences, San Diego) were added to cell suspension, mixed well, and reacted at room temperature for 15 min in the dark. Various data analyses of the flow cytometer were performed using CELLQuest software to adjust the FSC, SSC, and voltage; adjust the fluorescence compensation according to the standard fluorescein; and set the gate to detect the percentage of positive cells.

### 2.4. Lentiviral Transfection

The main chain of lentivirus vector, pGreenPuro shRNA (pGP), was purchased from Novobio Scientific Co., Ltd., Shanghai, China. Firstly, 293T cells (System Biosciences, USA) were transiently transfected with pGP Sir and pGP null to produce a large number of viruses with the use of pPACKH1 HIV lentivirus packaging kits (System Biosciences, USA). Secondly, the virus supernatant was isolated and purified and was then added to the 3rd to 6th generations BMSCs for 72 hours. The lentivirus was transfected under different multiplicity of infection (MOI) values (10, 30, 60, 80, and 100), and the most suitable MOI value was determined by immunofluorescence. Finally, the lentivirus genome was integrated into the BMSCs genome by virus transfection with MOI = 80. The transfection efficiency was detected by immunofluorescence. BMSCs were divided into two groups: (1) BMSCs transfected with empty lentivirus vectors (BMSC^Con^) and (2) BMSCs transfected with CXCR4 lentivirus vectors (BMSC^CXR4^).

### 2.5. Exosome Isolation and Identification

After 24 h of incubation in a serum-free medium, the BMSC supernatant was collected and subjected to 300g for 10 min, 3000g for 20 min, 10,000g for 30 min, and 100,000g for 2 h of ultracentrifugation at 4°C. Exosomes were obtained and stored at -80°C.

The exosomes were resuspended in PBS and were spread evenly over the copper grid. Then, they were negatively stained with the phosphotungstic acid for 10 min and were observed using a transmission electron microscopy.

The number and size of exosomes were assessed using the NanoSight NS300 system (Malvern Instruments, UK). The supernatant was diluted in PBS, and 1 ml of the solution was used for NanoSight analysis. Each sample was analyzed for 5 times, 10 s for each time. Western blot was used to detect the expression of Alix, TSG101, and CD9 proteins of the exosomes.

### 2.6. Cell Uptake of Exosomes

The exosomes were labeled with PHK26 (Sigma, USA) and were incubated with bEnd.3 in DMEM supplemented with 10% FBS for 48 hours. Then, they were washed with PBS to stop the cell absorption and fixed in 4% paraformaldehyde. Finally, the outcome of cell absorption was observed using the Olympus BX41 microscope equipped with a CCD camera.

### 2.7. Quantitative Reverse Transcription Polymerase Chain Reaction (qPCR)

Total RNA of BMSCs transfected with lentiviral was isolated using Trizol reagent (Invitrogen, United States). RNA samples from each group were reverse transcribed into cDNA using the PrimeScript™ RT reagent Kit (TAKARA, Japan). qPCR was performed on a Light Cycler thermal cycler system (Bio-Rad, United States) using SYBR Premix Ex Taq™II (TAKARA, Japan) and gene-specific primers. Gene-specific primers used are as follows: CXCR4—forward 5′-CGCAAATGGGCGGTAGGCGTG-3′ and reverse 5′-CATAGCGTAAAAGGAGCAACA-3′, and GAPDH—forward 5′-CCGCATCT TCTTGTGCAGTG-3′ and reverse 5′-ACCAGC TTCCCATTCTCAGC-3′.

### 2.8. Middle Cerebral Artery Occlusion in Rats

Twenty-four male adult SD rats weighing 250-270 g were divided into 4 groups with 6 rats in each group: the sham-operated group (sham group), the acute middle cerebral artery occlusion model (MCAO) rats injected with PBS group (control group), the MCAO rats injected with exosomes derived from BMSC^Con^ group (Exo group), and the MCAO rats injected with exosomes derived from BMSC^CXCR4^ group (Exo^CXCR4^ group). Rats were anesthetized with 1% pentobarbital intraperitoneally and then received an operation according to the modified Zea Longa method [26]. Briefly, a 6-0 coated with silicon nylon monofilament suture (Beijing Cinontech Co., Ltd., Beijing, China) was introduced into external carotid artery lumen and then gently advanced into the internal carotid artery in order to block the origin of the middle cerebral artery. For the rats in the sham-operated group, the suture was inserted into external carotid artery lumen but not advanced into the internal carotid artery. After 90 min occlusion, the suture was withdrawn to allow the reperfusion. A 5-point scale [26] was used to assess the neurological deficiency score, and the rats with a score between 1 and 3 were considered as a success of MCAO.

### 2.9. Exosome Transplantation

Exosome transplantation was conducted at 24 h after the establishment of MCAO. The injection was performed as described previously [27]. At a speed of 5 *μ*l/min, 100 *μ*g Exo, Exo^CXCR4^, or the same volume of PBS was injected into the lateral ventricle of the affected hemisphere (*X* = −0.5 mm, anterior-posterior; *Y* = 1.5 mm, medial-lateral; and *Z* = −3.5 mm, dorsal-ventral). The observation of vocalization, seizures, hemiplegia, and body weight was continued after surgery.

### 2.10. Triphenyl Tetrazolium Chloride (TTC) Staining

Rats were anesthetized with 2% pentobarbital intraperitoneally at 3 d after ischemia/reperfusion (I/R), and the brains were removed after profusion with NS. A brain slicer was used to coronally section the brains at 2 mm intervals from the frontal pole. The slices were incubated with 1% TTC solution for 15 minutes at 37°C in the dark and then were immersed with 4% paraformaldehyde.

### 2.11. Modified Neurological Severity Score

Rats were examined with modified neurological severity score (mNSS) at 1 d, 3 d, 7 d, 14 d, and 21 d after I/R. This evaluation was performed by a blinded tester. mNSS consists of motor (muscle status, abnormal movement), sensory (visual, tactile, and proprioceptive), reflex, and balance test. Scores ranged from 0 to a maximum of 14. The extent of neurological deficits was assessed as follows: 1-5 scores, mild deficits; 6-9 scores, moderate deficits, and 10-14 scores, severe deficits.

### 2.12. Adhesive Removal Test

The adhesive remove test was used to assess the integration of motor and sensory of rats at 1 d, 3 d, 7 d, 14 d, and 21 d after I/R. This evaluation was performed by a blinded tester. For each rat, an adhesive paper with the size of 1 × 1 cm was pasted on the surface of its forelimb plantar, and the time of removing the adhesive paper was recorded.

### 2.13. CCK8 Detects Cell Proliferation

The bEnd.3 cells were seeded in a 96-well plate at a density of 1 × 10^4^ cells per well, and 5 wells were set as one group. In the experiment of exosomes promoting the proliferation of bEnd.3 cells, the bEnd.3 cells were divided into 5 groups according to the concentrations of exosome (0, 5, 10, 15, and 20 *μ*g/ml); in the experiment of cobalt chloride inhibiting the proliferation of bEnd.3 cells, the bEnd.3 cells were divided into 5 groups according to the concentrations of cobalt chloride (0, 100, 200, 400, and 600 *μ*mol/l). Fresh medium containing 10% CCK8 solution was added into the wells and cultured in a 5% CO_2_-saturated humidity incubator at 37°C for 0.5 to 4 h. The absorbance value (OD value) of bEnd.3 cells at a wavelength of 450 nm was detected by a microplate reader.

### 2.14. Edu Staining Assay Detects Cell Proliferation

The bEnd.3 cells were divided into 4 groups: the bEnd.3 cells cultured with exosomes derived from BMSC^Con^ group (Exo^Con^ group); the bEnd.3 cells cultured with exosomes derived from BMSC^CXCR4^ group (Exo^CXCR4^ group); the bEnd.3 cells cultured with exosomes derived from BMSC^CXCR4^ and AMD3100 group (Exo^CXCR4^ + AMD3100 group), and the bEnd.3 cell control group. 50 *μ*mol/l Edu was added to the medium of bEnd.3 cells in each group and then cultured in a 5% CO_2_ incubator at 37°C. All cell nuclei were stained blue, while the proliferating cell nuclei were also stained red, which were quantified by the fluorescence microscope.

### 2.15. Cell Migration Assay

Cell migration was measured by a Transwell cell chamber. Conditioned medium with 15 *μ*g/ml Exo^Con^ or Exo^CXCR4^ was added to the lower chamber, while 1 × 10^5^ bEnd.3 cells were cultured in the upper chamber. After 24 h, the cells were fixed with formaldehyde and then stained with crystal violet (Sigma, USA). Migratory bEnd.3 cells were quantified by a microscope.

### 2.16. Tube Formation Assay

The bEnd.3 cells were incubated with a medium containing exosome or not for 24 h were collected. Cells in each group were seeded in 48-well plates (5 × 10^3^ cells per well) coated with matrix glue, and each group was established in three holes. After 6 h, the vascular tubular morphogenesis was observed in at 100x magnification by the inverted optical microscope. The number of vascular lumen structures in 5 random visual field was recorded by ImageJ software.

### 2.17. Western Blot

The total protein of the cells or exosomes was separated by SDS-PAGE. After electrophoretic transfer to polyvinyldifluoridene (PVDF) membranes (Millipore, Billerica, MA), the proteins were treated with SQSTM1/p62 (D1Q5S) (1 : 1000 dilution; CST, Boston), LC3A/B (D3U4C) XP (1 : 1000 dilution; CST, Boston), BAX (1 : 1000 dilution; Abcam, Cambridge, MA), CXCR4 (1 : 1000 dilution; Abcam, Cambridge, MA), *β*-tubulin (1 : 1000 dilution; Sigma, USA), TSG101 (1 : 1000 dilution; CST, Boston), CD9 (1 : 1000 dilution; Abcam, Cambridge, MA), Alix (1 : 1000 dilution; CST, Boston), Bcl2 (1 : 1000 dilution; Abcam, Cambridge, MA), PARP (1 : 1000 dilution; CST, Boston), Wnt-3a (1 : 1000 dilution; Wanleibio, Shenyang), *β*-catenin (1 : 1000 dilution; Millipore, Billerica, MA), and *β*-actin (1 : 1000 dilution; Proteintech, Chicago) overnight at 4°C followed by a horseradish peroxidase-linked secondary anti-mouse or anti-rabbit antibody (1 : 5000 dilution; ICL Lab, Portland Oregon). Finally, the antigen-antibody complexes were photographed by Pierce ECL Western Blotting Substrate.

### 2.18. Statistical Analysis

SPSS 20.0 software (IBM Corporation, NY, USA) and GraphPad Prism 7.0 (GraphPad Software Inc., CA, USA) were used for data analyses; all data were expressed as the mean ± standard deviation (SD). All sample data conformed to the normal distribution by the Kolmogorov-Smirnov test (*P* > 0.05). Differences between multiple groups were analyzed by one-way analysis of variance (ANOVA) test, and then, multiple comparisons were performed using Bonferroni post hoc test. Values of *P* < 0.05 are considered statistically significant.

## 3. Results

### 3.1. Identification of BMSCs and Exosomes

**Figure 1:**
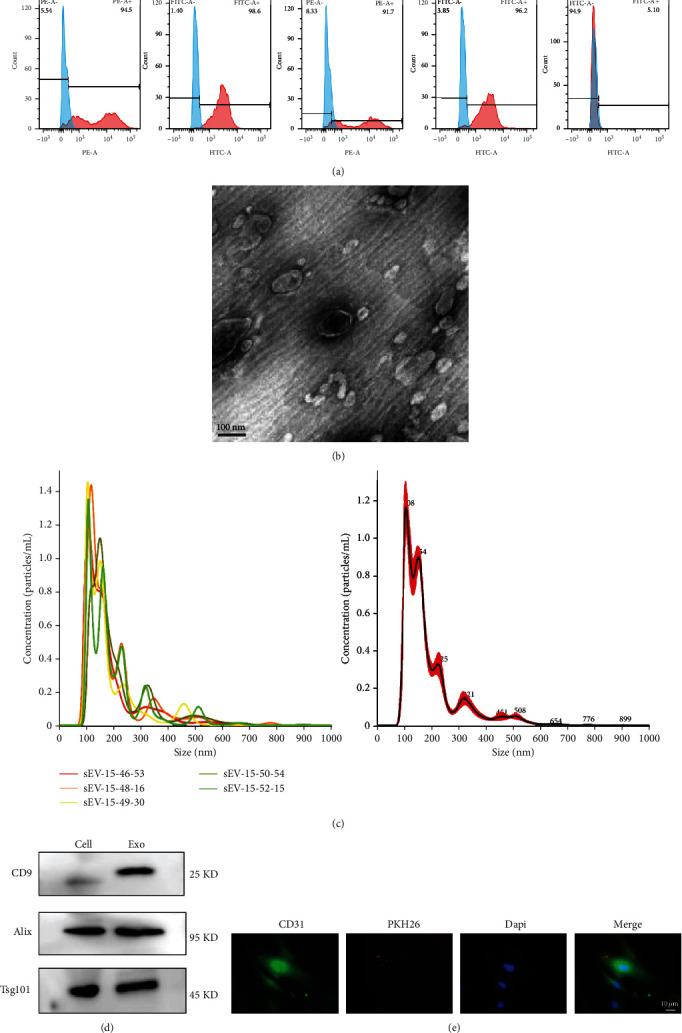
Identification of BMSCs and BMSC exosomes. (a) Identification of BMSC markers CD105(+), CD44(+), CD73(+), CD90(+), and CD34(-) by flow cytometry. (b) Transmission electron microscopy results for exosome ultrastructure. Scale bar = 100 nm. (c) Nanometer diameter range, average size, and concentration of the exosomes were detected by NanoSight. (d) Exosomes were isolated from normalized volumes of BMSC supernatants after 24 h of cell culture. Immunoblotting showed that the isolated exosome expressed CD9, TSG101, and Alix. (e) CD31-labeled bEnd.3 cells (green) were incubated with PKH26-labeled Exo (red) for 2 h, and Exo were swallowed by the bEnd.3 cell. Scale bars = 10 *μ*m.

BMSCs were isolated from SD rats, and the expression of immune markers in the third generation of them was identified by flow cytometry. As shown in Figure 1(a), the CD105 (94.5%), CD44 (98.6%), CD73 (91.7%), and CD90 (96.2%) were found to be positive, while the CD34 (5.10%) were found to be negative, which indicated the high purity of BMSCs.

The shape of the exosomes derived from BMSCs was a classic cup shape under transmission electron microscopy (Figure 1(b)). NTA tracking was used to analyze the size of exosomes, which indicated that the diameter distribution ranged from 50 nm to 600 nm, with a peak at 100 nm (Figure 1(c)). In addition, the expression of exosome protein markers was detected by Western blot. As shown in Figure 1(d), CD9, TSG101, and Alix were expressed in the exosomes. When exosomes labeled with the fluorescent PKH26 were incubated with bEnd.3 cells, the recipient cells—bEnd.3 cells—exhibited high uptake efficiency, as demonstrated by a fluorescence microscopy (Figure 1(e)).

### 3.2. Increased Expression of CXCR4 in Exosomes Derived from BMSCs Transfected with Lentivirus

**Figure 2:**
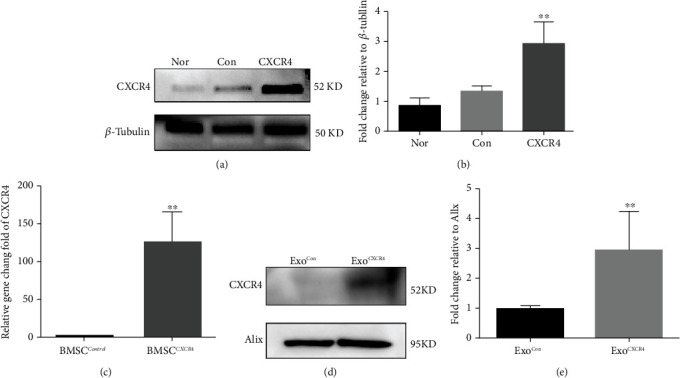
CXCR4 overexpression by BMSCs and exosomes. (a) Western blot results for expression of CXCR4 protein in BMSCs under normal conditions, transfection of control virus, and overexpression of CXCR4. (b) Expression statistics for CXCR4 proteins in BMSCs under normal conditions, transfection of control virus, and overexpression of CXCR4. (c) qPCR was used to detect mRNA after transfection of control virus and CXCR4-encoded virus. (d) Western bolt results for exosome expression. (e) Statistics for exosome CXCR4 protein expression. ^∗^*P* < 0.05, ^∗∗^*P* < 0.05, and ^∗∗∗^*P* < 0.01 vs. the control group.

The lentivirus conveying control vector and CXCR4-overexpressing vector were constructed and transfected into BMSCs. Compared with control groups, the protein and RNA expression of CXCR4 increased in the BMSCs which were transfected with lentivirus encoded by CXCR4 (Figures 2(a)–2(c)). As shown in Figures 2(d), the expression of Alix protein was similar between the Exo^Con^ and Exo^CXCR4^ groups, which indicated the similar concentration of exosome in both groups. Compared with the Exo^Con^ group, the expression of CXCR4 protein was significantly increased in the Exo^CXCR4^ group (*P* < 0.05, Figure 2(e)).

### 3.3. Injection of Exo^CXCR4^ Improved Neurobehavioral Outcome in MCAO Rats

**Figure 3:**
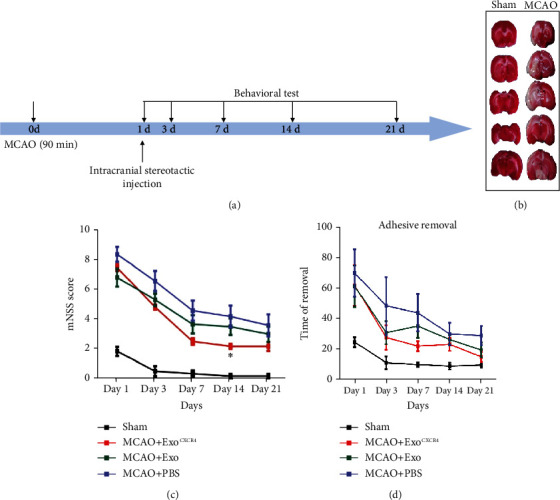
BMSC^CXCR4^-derived exosomes improved neurobehavioral outcome in MCAO rats. (a) Experimental design. Middle cerebral artery was blocked for 90 minutes; 100 *μ*g of exosomes or the same volume of PBS was stereotactically injected into the ipsilateral lateral ventricle of a rat's brain; (b) TTC staining for rats in sham and MCAO groups (*n* = 6); (c) mNSS score for rats in each group (*n* = 6); (d) times of adhesive removal from rats' right paws in each group (*n* = 6).

The neurobehavior outcomes of the rats were measured for day 1, day 3, day 7, day 14, and day 21 (Figure 3(a)). We measured the cerebral infarction volume of the rats for day 3 by TTC staining in both the sham and MCAO groups and found that the cerebral infarct volumes were significantly different among the 2 groups (*n* = 6, Figure 3(b)). As shown in Figure 3(c), compared with the control group and Exo^Con^ group, the Exo^CXCR4^ group showed a significantly lower mNSS score at 14 d after I/R (*P* < 0.05, *n* = 6, Figure 3(c)). For the time of removing sticky papers, there was no significant difference among each groups (*P* > 0.05, *n* = 6, Figure 3(d)).

### 3.4. The Role of Exo^CXCR4^ in the Proliferation, Migration, and Tube Formation of bEnd.3 Cells

**Figure 4:**
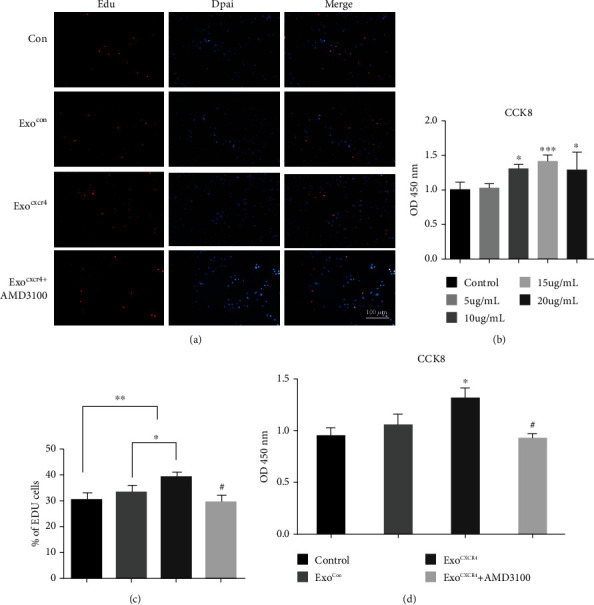
Effect of BMSC^CXCR4^-derived exosomes on endothelial cell proliferation. (a) Edu immunofluorescence staining; (b) CCK8 detects the effects of different exosomes concentrations on proliferation; (c) Edu cell count; (d) CCK8 detects the proliferation for each group. ^∗^*P* < 0.05, ^∗∗^*P* < 0.05, and ^∗∗∗^*P* < 0.01 vs. the control group. Scale bars = 100 *μ*m.

**Figure 5:**
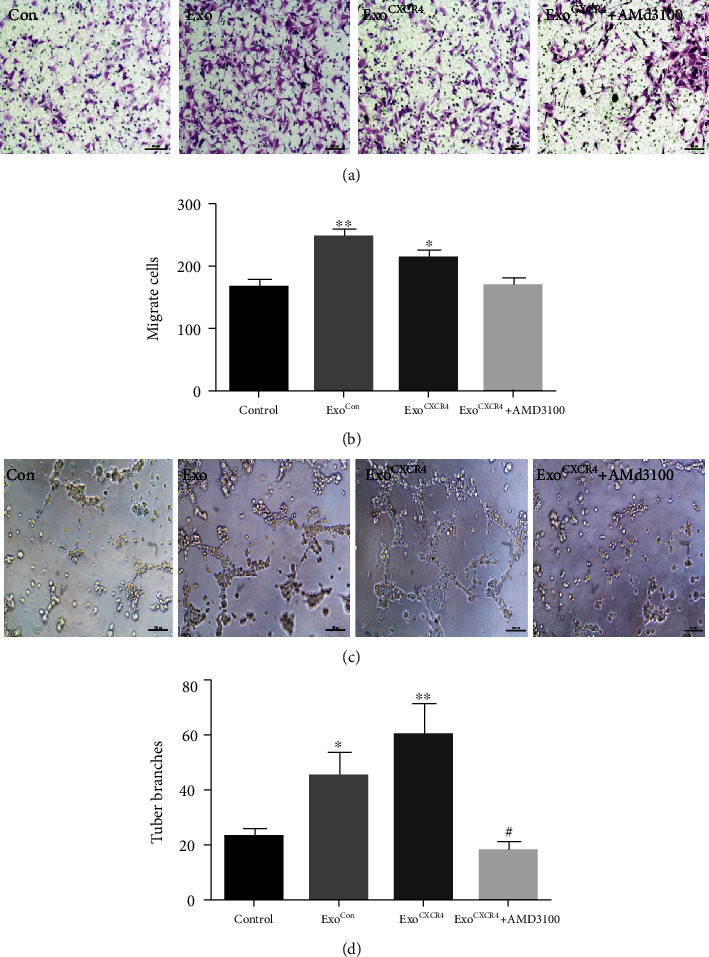
Effect of BMSC^CXCR4^-derived exosomes on endothelial cell migration and lumen formation. (a) Transwell assay detects the effect of exosomes on migration; (b) quantitative migration data for each group on migration; (c) exosome effect on angiogenesis; (d) quantitative data for each group's effect on angiogenesis. ^∗^*P* < 0.05, ^∗∗^*P* < 0.05, and ^∗∗∗^*P* < 0.01 vs. the control group.

Exo^Con^ were added to bEnd.3 cells. It was found that the proliferation of bEnd.3 cells was enhanced with increasing exosome concentrations. The proliferative ability of bEnd.3 cells reached its peak at the exosome concentration of 15 *μ*g/ml, which was used in the following experiments (Figure 4(b)). Then, bEnd.3 cells were cultured with Exo^Con^, Exo^CXCR4^, or Exo^CXCR4^ plus AMD3100, and the Edu staining and CCK8 were used to detect the proliferation of bEnd.3 cells. As shown in Figures 4(a), 4(c), and 4(d), the bEnd.3 cells in the Exo^CXCR4^ group have stronger proliferation ability than other groups (*P* < 0.05), while the CXCR4 inhibitor (AMD3100) can reduce this effect. As for the migration of bEnd.3 cells, Exo^Con^ can significantly promote the migration compared with the control group (*P* < 0.05), while there was no significant difference between the Exo^CXCR4^ and Exo^Con^ groups (*P* > 0.05, Figures 5(a) and 5(b)). As indicated in Figures 5(c) and 5(d), compared with the control group, Exo^Con^ can promote the angiogenesis of the endothelium (*P* < 0.05). Additionally, the Exo^CXCR4^ can further promote the angiogenesis of the endothelium compared with the Exo^Con^ group (*P* < 0.05). The addition of AMD3100 will seriously affect the tube formation.

### 3.5. Exo^CXCR4^ Can Reduce Cell Apoptosis Caused by Cobalt Chloride (COCl_2_)

**Figure 6:**
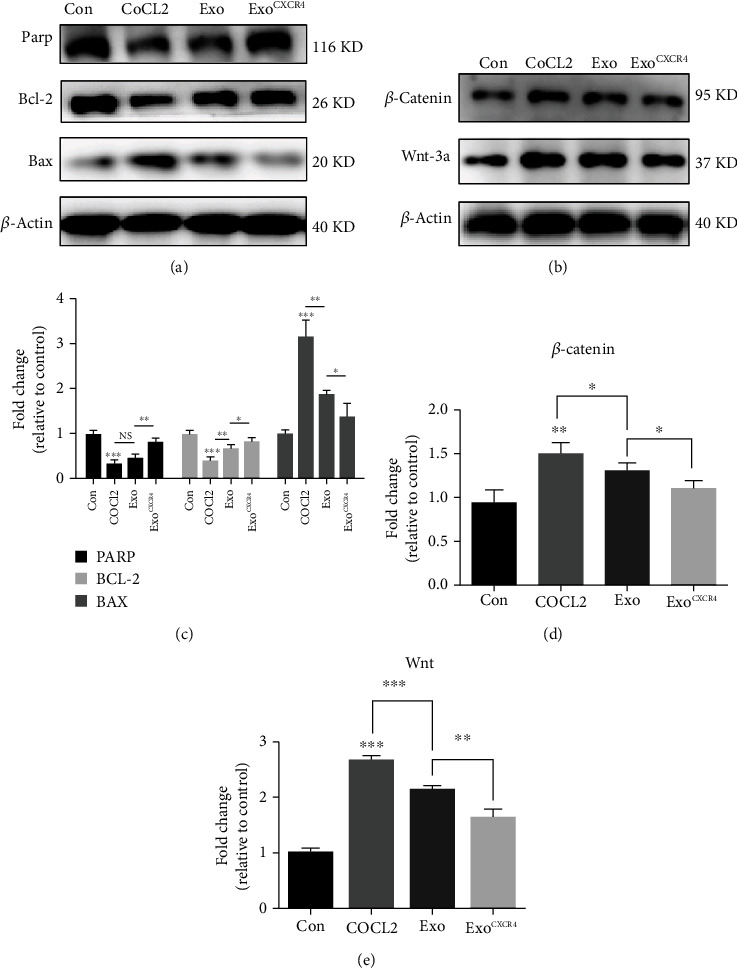
Effect of exosomes derived from BMSC^CXCR4^ on apoptosis. (a) Western blot was used to examine the effect of each group on apoptosis induced by COCl2; (b) *β*-catenin and Wnt-3a expression level in bEnd.3 cells was analyzed by Western blot; (c–e) statistics for each group. ^∗^*P* < 0.05; ^∗∗^*P* < 0.01.

Western blot was used to assess the expression of the proapoptotic protein BAX and antiapoptotic proteins Bcl-2 and PARP. As shown in Figures 6(a) and 6(c), compared with the control group, COCl_2_ can significant increase the expression of BAX and reduce the expression of Bcl-2 and PARP (*P* < 0.05). Compared with the COCl_2_ group, the expression of Bcl-2 and PARP in the COCl_2_ + Exo^Con^ group was higher, while the expression of BAX was lower (*P* < 0.05). In addition, Exo^CXCR4^ can further reduced cell apoptosis caused by COCL_2_.

Finally, the potential mechanism of antiapoptosis effect of Exo^CXCR4^ was detected by Western blot. As shown in Figures 6(b), 6(d), and 6(e), COCl_2_ can increase the expression of *β*-catenin and Wnt-3, while Exo^Con^ can reduce the expression of these proteins. Additionally, Exo^CXCR4^ can further attenuate the activation of the Wnt-3a/*β*-catenin pathway. This indicated that the antiapoptosis effect of Exo^CXCR4^ was related to the Wnt-3a/*β*-catenin pathway.

## 4. Discussion

The present study takes advantage of the important role of the CXCL12/CXCR4 axis in the recovery of cerebral infarction. The expression of CXCL12 was significantly upregulated after cerebral ischemia, leading to chemotaxis of endothelial progenitor cells expressing CXCR4 [28]. Additionally, hours after cerebral infarction, CXCL12 expression was elevated in both astrocytes and endothelial cells [29]. Compared to CXCR7, another receptor for CXCL12, CXCR4, is mostly expressed on the cell membrane [30]. Exosomes are vesicles that are packaged by the cell membrane for information transmission [31]. Therefore, it was hypothesized that by increasing CXCR4 on the cell membranes, the amount of CXCR4 on exosomes could be increased. In the present study, BMSCs were genetically modified to overexpress CXCR4 with the help of lentiviral transfection and the exosomes secreted by BMSCs were isolated. According to the results of Western blot and qPCR, CXCR4 was detectable on the Exo^CXCR4^ group, while very little CXCR4 expression was found on Exo^Con^ from BMSCs transfected with no-load virus. Additionally, the recipient cells, bEnd.3 cells, exhibited high uptake efficiency of exosomes as demonstrated by a fluorescence microscopy. These results provided foundation for the research of exosomes in *vivo* and *vitro*.

In *in vivo* experiments, TTC staining was firstly used to assess the cerebral infarction volume of the rats for day 3 to make sure the success of the conduction of MCAO model. Then, the mNSS score and adhesive removal test were used to assess the recovery of the MCAO rats. The results of these tests shown that the injection of exosomes can accelerate the recovery of the MCAO rats, and the rats in the Exo^CXCR4^ group recovered the fastest. This indicated that CXCR4 might promote the positive effect of exosomes in ischemia reperfusion injury.

In *in vitro* experiments, Exo^CXCR4^ were able to promote the proliferation and tube formation of brain microvascular endothelial cells. These effects were all offset by AMD3100, a CXCR4 inhibitor. This evidence does not only prove that BMSC exosomes can promote the angiogenesis in brain tissues after ischemia but also indicates that CXCR4 can improve this effect. However, Exo^CXCR4^ did not promote cell migration better than ordinary BMSC exosomes. This may be because the taken-up exosomes did not completely transfer the CXCR4 to the cell membrane. Besides, the CXCR4 content transferred by exosomes is obviously lower than the CXCR4 content of the cell surface itself, so the total amount of CXCR4 on the cell surface did not change significantly.

BMSCs have a significant inhibitory effect on inflammation. Previous studies have injected BMSCs into animals with central nervous system damage and found that BMSCs can survive and migrate to damaged tissues and ultimately improve neural function [15, 32]. The CXCL12/CXCR4 axis plays an important role in the treatment of ischemic stroke with transplanting BMSCs [33]. Previous studies have shown that expression of CXCL12 in astrocytes is upregulated within 7 days after cerebral ischemia in newborn mice [29]. CXCR4 is expressed on the surface of BMSCs, which may be an important reason that helps BMSCs migrate to the ischemic area. However, most intravascularly injected BMSCs migrate to the lungs, probably due to insufficient expression of CXCR4 in BMSCs [34, 35]. Therefore, many studies have attempted injection of BMSCs with high expression of CXCR4 or stereotactic injection of BMSCs [36, 37]. Different from BMSCs, exosomes were small with low immunogenicity. It is not easy for exosomes to block blood vessels and easy to store [25, 38]. They are now increasingly used for the exploration of various diseases. Exosomes have been introduced into regenerative medicine as an alternative to BMSCs [39]. However, how to efficiently deliver exosomes to the ischemic area remains unclear. Similar to BMSCs, there are only a few CXCR4 on the surface of exosomes. Most of the exosomes circulate in the body during intravenous injection and their utilization efficiency is uncertain [40]. Therefore, in the present study, exosomes were derived from BMSCs which were transfected with lentivirus encoded by CXCR4. *In vivo* experiments determined that functional recovery of rats in the Exo^CXCR4^ group was significantly improved compared with rats injected with ordinary exosomes.

After blood clot formation during ischemic stroke, endothelial cells undergo apoptosis due to lack of nutrients, which will destroy the blood-brain barrier, cause brain tissue edema, and aggravate disease progression [41, 42]. The promotion of new vessels in the ischemic region is important for improving the outcome of cerebral infarction. Data have shown that in the 21 days after ischemia, new blood vessels are formed at the edge of the ischemic area, which gradually extends to the ischemic center through budding [9]. Previous studies have shown that CXCL12/CXCR4 can promote the stabilization of endothelial cells and improve the outcome of atherosclerotic diseases by antiapoptotic *in vivo* protein [43]. COCl_2_ can cause hypoxia in cells, leading to autophagy and apoptosis of cells [44]. In the present study, COCl_2_ can significantly increase the expression of proapoptotic protein BAX and reduce the expression of antiapoptotic proteins Bcl-2 and PARP, while Exo^Con^ can reverse this effect caused by COCl_2_. Exo^CXCR4^ can further reduce cell apoptosis caused by COCL_2_. Additionally, COCl_2_ can increase the expression of *β*-catenin and Wnt-3, while Exo^Con^ can reduce the expression of these proteins. Exo^CXCR4^ can further attenuate the activation of the Wnt-3a/*β*-catenin pathway. Thus, the present study shows that Exo^CXCR4^ can promote the proliferation and tube formation of microvascular endothelial cells and plays an antiapoptotic role via the Wnt-3a/*β*-catenin pathway, which is consistent with the previous results.

However, this study mainly investigated the effect and mechanism of exosomes derived from BMSCs in promoting angiogenesis in ischemia/reperfusion injury, without further research into the specific components of BMSC exosome by which promoted angiogenesis. This is the limitation in this study as well as our future research directions.

## 5. Conclusion

In ischemia/reperfusion injury, Exo^CXCR4^ can promote the proliferation and tube formation of microvascular endothelial cells and play an antiapoptotic role via the Wnt-3a/*β*-catenin pathway.

## Data Availability

The raw data supporting the conclusions of this manuscript will be made available by the authors, without undue reservation, to any qualified researcher.
